# Mouthwash Effects on LGG-Integrated Experimental Oral Biofilms

**DOI:** 10.3390/dj8030096

**Published:** 2020-09-01

**Authors:** Qingru Jiang, Veera Kainulainen, Iva Stamatova, Sok-Ja Janket, Jukka H. Meurman, Riitta Korpela

**Affiliations:** 1 Department of Oral and Maxillofacial Diseases, University of Helsinki and Helsinki University Hospital, 00100 Helsinki, Finland; iva.stamatova@abv.bg (I.S.); jukka.meurman@helsinki.fi (J.H.M.); 2 Department of Pharmacology, Faculty of Medicine, University of Helsinki, 00100 Helsinki, Finland; veera.kainulainen@helsinki.fi (V.K.); riitta.korpela@helsinki.fi (R.K.); 3 Human Microbiome Research Program, Faculty of Medicine, University of Helsinki, 00100 Helsinki, Finland; 4 Translational Oral Medicine, Forsyth Institute, Cambridge, MA 02142, USA; sjanket@forsyth.org

**Keywords:** probiotic-related biofilm, antimicrobial effect, biofilm age, biofilm recovery

## Abstract

In order to investigate the effects of mouthwashes on oral biofilms with probiotics, we compared in biofilms the susceptibility to mouthwashes of probiotic *Lactobacillus rhamnosus* GG (LGG) and oral pathogens *Streptococcus mutans*, *Streptococcus sanguinis*, and *Candida albicans*. We also evaluated these pathogens’ susceptibility to the mouthwashes and their recovery after mouthwash-rinsing in biofilms with/without LGG. First, 1-day-/3-day-old LGG-integrated multi-species biofilms were exposed for 1 min to mouthwashes containing chlorhexidine, essential oils, or amine fluoride/stannous fluoride. Cells were plate-counted and relative survival rates (RSRs) of LGG and pathogens calculated. Second, 1-day-/3-day-old multispecies biofilms with and without LGG were exposed for 1 min to mouthwashes; cells were plate-counted and the pathogens’ RSRs were calculated. Third, 1-day-old biofilms were treated for 1 min with mouthwashes. Cells were plate-counted immediately and after 2-day cultivation. Recovery rates of pathogens were calculated and compared between biofilms with/without LGG. Live/Dead^®^ staining served for structural analyses. Our results showed that RSRs of LGG were insignificantly smaller than those of pathogens in both 1-day and 3-day biofilms. No significant differences appeared in pathogens’ RSRs and recovery rates after treatment between biofilms with/without LGG. To conclude, biofilm LGG was susceptible to the mouthwashes; but biofilm LGG altered neither the mouthwash effects on oral pathogens nor affected their recovery.

## 1. Introduction

Dental caries and periodontal diseases are preventable biofilm-associated diseases. The total burden of those diseases increases globally as more elderly people retain a greater number of their teeth. Oral biofilms are present as diverse communities of bacteria and fungi embedded in a highly specialized extracellular matrix. Biofilm formation is a dynamic and continuous process involving initial reversible attachment, irreversible attachment, maturation, and dispersion [1]. Good oral self-care practice is essential in reducing the accumulation of oral biofilms (dental plaque), and controlling/reducing the risks of dental diseases [2,3,4]. Manual and powered toothbrushes to various degrees reduce plaque and gingivitis in the short and long term [5]. Although mechanical cleaning is crucial to prevent oral diseases, oral biofilms are nearly impossible to remove completely, such as those located in fissures, buccal pits, posterior interproximal areas, and gingival margins, where dental pathologies mostly develop [6,7]. The use of mouthwashes, a chemical cleaning of the teeth, is usually meant to support mechanical cleaning to reduce accumulation of dental plaque; it can even serve as the only oral care for those patients unable to brush their teeth, either after surgery or because of motor or cognitive limitations [8]. Among the many antiseptic components of oral mouthwashes, chlorhexidine (CHX) has long been considered the gold standard for short-term use, acting on bacteria, spores, and fungi [8,9,10,11,12]. When used as an adjunct to toothbrushing for 1 to 6 months, CHX dentifrices significantly reduce plaque accumulation and alleviate signs and symptoms of gingival inflammation [13,14]. However, prolonged use of CHX in most of the cases leads to large increases in extrinsic tooth staining. Essential oils (EOs) and amine fluoride/stannous fluoride (AmF/SnF_2_) have been suggested as effective alternatives to CHX as adjuncts to oral hygiene [8,15].

Bacteriotherapy emerges as an appealing alternative in combating pathogenic biofilms. Probiotics, “live microorganisms that, when administered in adequate amounts, confer a health benefit on the host” [16], have shown favorable properties in maintaining oral health. Both in vitro and clinical studies support the role of healthy bacteria in reducing the risk of oral infectious diseases. The regular long-term intake of probiotic-supplemented milk may reduce caries development in high-caries-risk preschool children [17]. The true mechanisms still remain unclear, but it could be speculated that these can serve as competition for binding sites in developing biofilms, microbial antagonism, and quorum sensing. Probiotics *Lactobacillus rhamnosus* GG (LGG), *L. reuteri* ATCC 55730, *L. acidophilus* LA-5, *L. paracasei* F19, and *Bifidobacterium animalis* BB-12 are reported to be able to colonize in vivo in the oral cavity for the short term [18,19,20,21,22]. However, there is still insufficient evidence that extrinsic probiotics could permanently establish themselves in oral biofilms and participate in oral equilibrium. Thus, they may easily be eliminated from the mouth when oral hygiene practices are regularly performed. Previous studies have shown that CHX had no detectable effect on salivary *L. rhamnosus* [23], and *Lactobacillus* strains were less susceptible to CHX and sodium fluoride (NaF) than was *Streptococcus mutans* [24,25,26]. A probiotic with multiple health benefits, LGG was able to integrate into experimental biofilms [27]. A new concept has been introduced showing that probiotic mouthwashes could be alternatives to conventional mouthwashes with minimal side effects on gingival health [28]. Hence, we were interested to assess whether LGG survives when exposed to a mouthwash and to compare its survival with the survival of streptococci and *Candida* strains in biofilms.

Furthermore, we were interested in finding out whether the susceptibility of oral (opportunistic) pathogens in biofilms would change in the presence of probiotics. Biofilm micro-organisms are known to be more resistant to antimicrobial agents than those in planktonic cultures [29,30], which may be due to the complex physical, chemical, and biological properties of biofilms [31]. These effects can be further enhanced in microbial communities. Susceptible pathogens can be rendered resistant if neighboring, non-pathogenic cells produce a neutralizing or drug-degrading enzyme [32]. For example, beta-lactamase detected in gingival crevicular fluid can inactivate penicillin in the gingival sulcus, allowing the periodontal microbiota to survive [33]. We aimed to evaluate whether the addition of probiotics into multi-species biofilms would weaken the efficacy of the mouthwash against the oral pathogenic streptococci and *Candida*.

Bacterial cells may escape the effects of antimicrobials without undergoing genetic change; these cells, known as persisters, usually comprise about 1% in the stationary state and in biofilms [34]. We thus assumed that some persister cells of probiotics would remain in the biofilms after the mouthwash treatment. The administration of probiotics is known to be able to suppress the growth of oral pathogenic streptococci and *Candida* [27,35]. Consequently, we were interested in the recovery growth ability of oral pathogenic streptococci and *Candida* in the biofilms in the presence of the residual probiotic persister cells.

In the established experimental oral biofilms model [27], our first aim was to study the probiotic LGG’s susceptibility to mouthwashes compared with that of *S. mutans*, *S. sanguinis*, and *Candida albicans* (SSC) in the biofilms. Second, we investigated the influence of LGG on the efficacy of mouthwashes on SSC in the biofilms. Third, we studied the recovery of SSC in LGG-free and LGG-integrated biofilms after the mouthwash rinsing. Our hypothesis was that the probiotic strain differs from the oral pathogens in its susceptibility and function in the biofilm.

## 2. Materials and Methods

### 2.1. Strains, Growth Conditions, and Inoculum Preparation

This study employed the probiotic strain LGG ATCC 53103 (Valio Ltd., Helsinki, Finland) and five oral bacterial/yeast strains, namely *S. mutans* ATCC 27351, *S. sanguinis* ATCC 10556, *Aggregatibacter actinomycetemcomitans* ATCC 43718, *Fusobacterium nucleatum* ATCC 25586, and *C. albicans* ATCC 10231.

The details of the growth conditions and inoculum preparation have been described in a previous article [36]. In brief:

The strains, maintained as frozen stock, were cultivated twice on various agars, and a pure colony of each strain was inoculated and cultivated overnight in broth. For the strains and growth conditions see Table 1. For preparation of the cell suspensions, the centrifuged bacterial and yeast strains were washed three times with physiological saline (PS) and re-suspended in biofilm medium to below cell concentrations: LGG 1.64 × 10^8^ cells/mL, *S. mutans* 7.53 × 10^8^ cells/mL, *S. sanguinis* 3.31 × 10^8^ cells/mL, *A.actinomycetemcomitans* 4.44 × 10^9^ cells/mL, *F. nucleatum* 1.72 × 10^8^ cells/mL, and *C. albicans* 3.33 × 10^7^ cells/mL. Aliquots of the five species’ oral bacterial/yeast suspensions were then pooled for the preparation of LGG-free biofilms, and aliquots of all the six strains suspensions for LGG-integrated biofilms.

### 2.2. Preparation of Biofilms

The descriptions of the biofilms’ preparation appear in a previous article [36].

Biofilms were grown on vertically positioned saliva-coated hydroxyapatite (HA) discs (Clarkson Chromatography Products, Inc., South Williamsport, PA, USA). A salivary pellicle was formed by immersing an HA disc in processed and pasteurized whole saliva for 4 h at room temperature (RT). Then the saliva-coated HA discs were cultured with 0.3 mL of pooled strain suspension and 2.5 mL biofilm medium in 24-well plates anaerobically at 37 °C for 16.5 h or 64.5 h in the dark. The biofilm medium was renewed at the respective time points of 16.5 h and 40.5 h.

Ethics approval of this study is not applicable, as the whole saliva used in this study came from the pooled depot of the laboratory of the Department of Oral and Maxillofacial Diseases, University of Helsinki. Saliva was used only for coating the HA discs. No data through intervention or interaction with the individuals or identifiable private information were available regarding the saliva samples. Written informed consent came from all participants when the saliva for the pool was originally collected.

### 2.3. Susceptibility of Probiotic LGG to Mouthwashes Compared with Streptococci and Candida in the Biofilms

**Figure 1:**
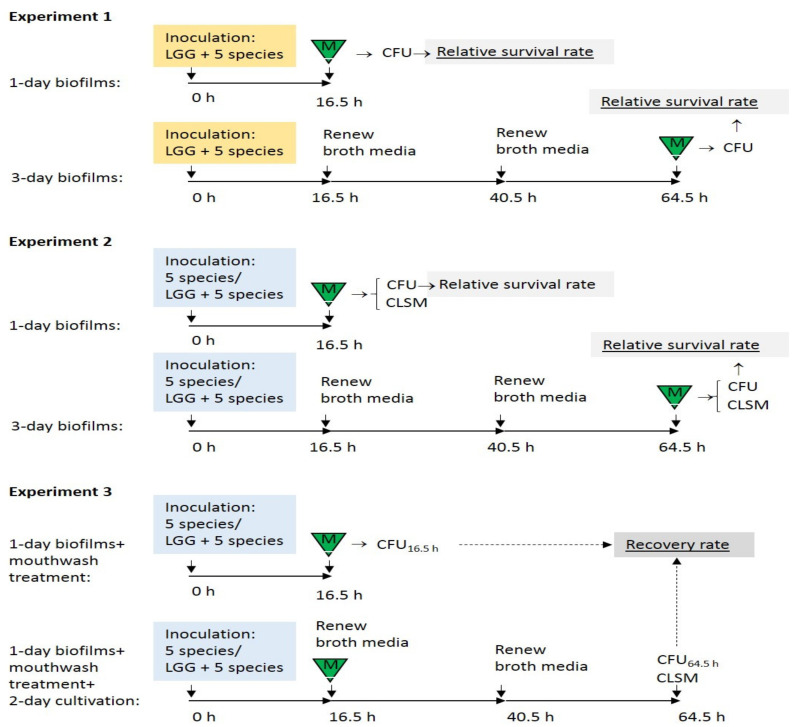
Study designs. Five species: a mixture of cell suspensions of *S. mutans*, *S. sanguinis*, *A. actinomycetemcomitans*, *F. nucleatum*, and *C. albicans*; LGG + 5 species: a mixture of cell suspensions of the above five species and probiotic LGG (*L. rhamnosus* GG); triangle-M: 1-min mouthwash exposure; CFU: plate count to obtain colony-forming units; CLSM: biofilm structure analysis with confocal laser scanning microscopy.

This experiment constructed 1-day and 3-day LGG-integrated oral biofilms (see Figure 1, Experiment 1 for aim 1).

After the cultivation, the biofilms were treated with mouthwash for 1 min. These commercial mouthwashes are in Table 2. The negative controls were the biofilms treated with PS. After the 1-min exposure in 1.8 mL mouthwash, each disc was rinsed in 1.8 mL PS for 5 min before the next step [37]. At the end of each design, biofilms were collected for enumeration of viable cells.

#### 2.3.1. Enumeration of Live Cells

After two dip washes in PS, each HA disc was transferred into 5 mL PS, and vigorously vortexed for 2 min (by Vortex-Genie^®^ 2 mixer, Scientific industries, Inc, Bohemia, NY, USA) followed by 5-sec sonication at RT (by Wagner Instrusonic, 90/180 watts, PS-Terä Oy, Lahti, Finland,). The sonicated cells were serially diluted and cultivated on brain heart infusion (BHI), Sabouraud, and de Man, Rogosa and Sharpe (MRS) agar plates at 37 °C in 5% CO_2_ for 48–72 h. colony-forming units (CFU) of LGG (from MRS plates), *S. mutans* (from BHI plates), *S. sanguinis* (from BHI plates), and *C. albicans* (from Sabouraud plates) were counted based on their colony morphology on the agar plates.

#### 2.3.2. Calculation of the Relative Survival Rates

Based on the viable cell numbers of LGG, of *S. mutans*, of *S. sanguinis*, and of *C. albicans* from the biofilms, the relative survival rate of each strain was calculated and compared. The relative survival rates were defined as viable cell numbers of strains in biofilms exposed to mouthwashes compared with that number exposed to PS. The relative survival rate of each strain was calculated using the following equation: relative survival rate = 100% × (CFU_mouthwash_)/(CFU_PS_).

### 2.4. Influence of LGG on the Efficacy of Mouthwashes on Biofilm Streptococci and Candida

This experiment constructed 1-day and 3-day LGG-free and LGG-integrated oral biofilms (see Figure 1, Experiment 2 for aim 2). Then the biofilms were treated with a single exposure of the mouthwash (PS/Corsodyl^®^/Listerine^®^/Meridol^®^) for 1 min. After the treatment, the biofilms were collected for enumeration of their viable cells. The relative survival rates of SSC were calculated and compared between the LGG-free and LGG-integrated biofilms. The mouthwash-treated biofilms were stained for structural analysis.

For the structural analysis of the biofilms, another copy of biofilms on saliva-coated HA discs treated with the mouthwashes was stained and scanned (see Figure 1). After two dip washes in PS, the biofilm samples were stained for 15 min in the dark at RT with the LIVE/DEAD *Bac*light^TM^ Bacterial Viability Kit (Molecular Probes^TM^, Life Technologies^TM^, Eugene, OR, USA), containing Syto 9 for live cells and propidium iodide for dead cells. Then all the biofilm samples were embedded in Mowiol mounting medium overnight at RT and were examined with an inverted confocal laser scanning microscope (CLSM) Leica SP8 (Leica Microsystems Gmbh Wetzlar, Germany). CLSM images were obtained with a ×40 water immersion objective. Biofilms were scanned randomly as a z-series of optical sections (0.50 µm thick/section, 10 areas/disc). Software Fiji was used to process the digital images [38].

### 2.5. Recovery of Streptococci and Candida in LGG-Free and LGG-Integrated Biofilms after Mouthwash Rinsing

This experiment constructed 1-day (16.5 h) LGG-free and LGG-integrated oral biofilms (see Figure 1, Experiment 3 for aim 3). The biofilms were treated with single-exposure mouthwash (PS/Corsodyl^®^/Listerine^®^/Meridol^®^) for 1 min. One portion of the treated biofilms was immediately collected for counting viable cell numbers of SSC. The other portion of the mouthwash-treated biofilms was allowed to continuously grow for another 2 days (48 h). After the 2-day cultivation, the biofilms were collected for enumeration of viable cell numbers of SSC and stained for structural analysis.

Comparison of the specific growth rate of SSC during the following 2 days after the 1-min mouthwash exposure required knowledge of the recovery rates, defined as the apparent generation times of the attached counts during the 2 days after mouthwash exposure (see Figure 1). “Apparent” means that a combination of overall proliferation, and the balance between attached, detached, and regenerated cells was integrated into each count of viable attached cells [39]. Recovery rates were calculated by the following equation: recovery rate _PS/Corsodyl_^®^_/Listerine_^®^_/Meridol_^®^ = (ln (CFU_64.5h_)–ln (CFU_16.5h_)) 48h. CFU_64.5h_ is the viable cell number collected at the time point of 64.5 h. CFU_16.5h_ is the viable cell number collected immediately after the mouthwash treatment at the time point of 16.5 h. The recovery rates for SSC were calculated and compared between the LGG-free and LGG-integrated biofilms.

### 2.6. Statistical Analysis

All the experiments were carried out in triplicate, and averages were used. Statistical analyses were performed with both IBM SPSS Statistics version 22 for Windows and rechecked with SAS version 9.3 for Windows. To determine the statistical significance, non-parametric tests: independent-samples Kruskal–Wallis one-way ANOVA served for multiple comparisons, and independent samples Mann–Whitney U tests for two samples. A difference was deemed significant at *p <* 0.05.

## 3. Results

### 3.1. Susceptibility to Mouthwashes of Probiotic LGG Compared with Susceptibility of Streptococci and of Candida in the Biofilms

#### Relative Survival Rates of LGG, Streptococci, and Candida in LGG-Integrated Biofilms

**Figure 2:**
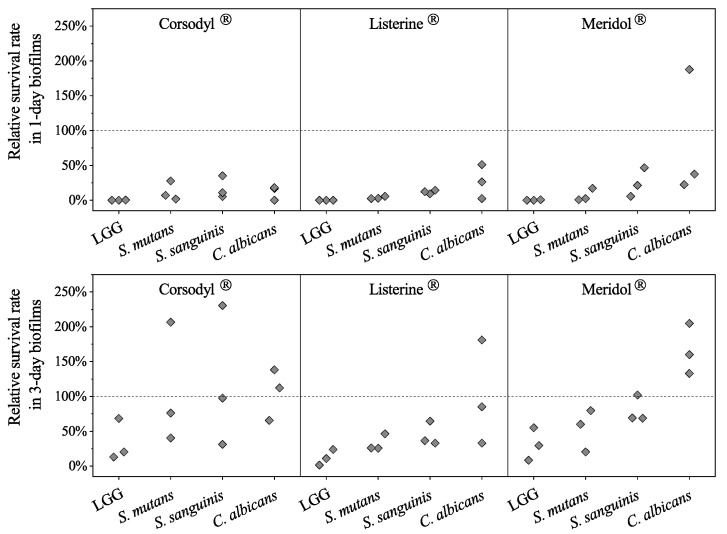
Relative survival rates of the strains in 1-day and 3-day LGG-integrated biofilms. Values as diamonds. Non-parametric tests: independent samples Kruskal–Wallis one-way ANOVA showed no significant differences (*p* > 0.05).

No significant differences emerged between LGG and the rest of the strains in the 1-day and 3-day biofilms treated with each of the mouthwashes (Figure 2). The lowest median relative survival rates were with treatment with Corsodyl^®^: for LGG in 1-day and 3-day biofilms (0.18% and 20.38%), followed by *S. mutans* (7.06% and 76.27%), and *S. sanguinis* (10.98% and 97.58%), and *C. albicans* (16.82% and 112.15%); followed by Listerine^®^ treatment: with *S. mutans* (2.71% and 26.02%), *S. sanguinis* (12.23% and 36.56%), and *C. albicans* (26.53% and 85.25%); and followed by Meridol^®^ treatment: with *S. mutans* (2.57% and 60.13%), *S. sanguinis* (21.42% and 69.23%), and *C. albicans* (37.72% and 160.00%), (*p >* 0.05).

### 3.2. Influence of LGG on Mouthwash Efficacy against Streptococci and Candida in the Biofilms

#### 3.2.1. Relative Survival Rates of Streptococci and *Candida* in LGG-Free and LGG-Integrated Biofilms

**Figure 3:**
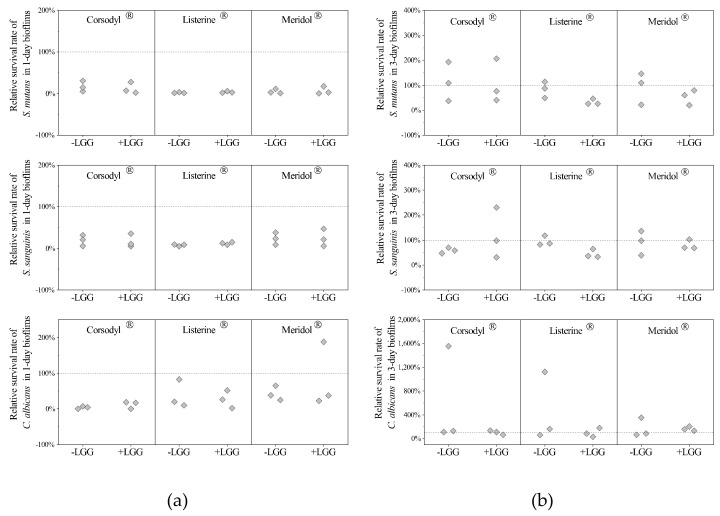
(**a**) Relative survival rates of the strains in 1-day LGG-free (-LGG) and LGG-integrated (+LGG) biofilms; (**b**) Relative survival rates of the strains in 3-day LGG-free (-LGG) and LGG-integrated (+LGG) biofilms. Values as diamonds. Dotted line indicates relative survival rate that equals 100%. The independent samples Mann–Whitney U test showed no significant differences (*p >* 0.05).

Between the LGG-free and LGG-integrated biofilms, no significant differences were detectable in the relative survival rates of SSC in 1-day or 3-day biofilms treated with each mouthwash (*p* > 0.05, Figure 3). The median relative survival rates of all the strains in 1-day biofilms after mouthwash treatments were all lower than the rates in the 3-day biofilms. The median relative survival rates of *C. albicans* in 3-day biofilms treated with Corsodyl^®^ were both above 100% (Figure 3).

#### 3.2.2. Structural Analysis of Biofilms

**Figure 4:**
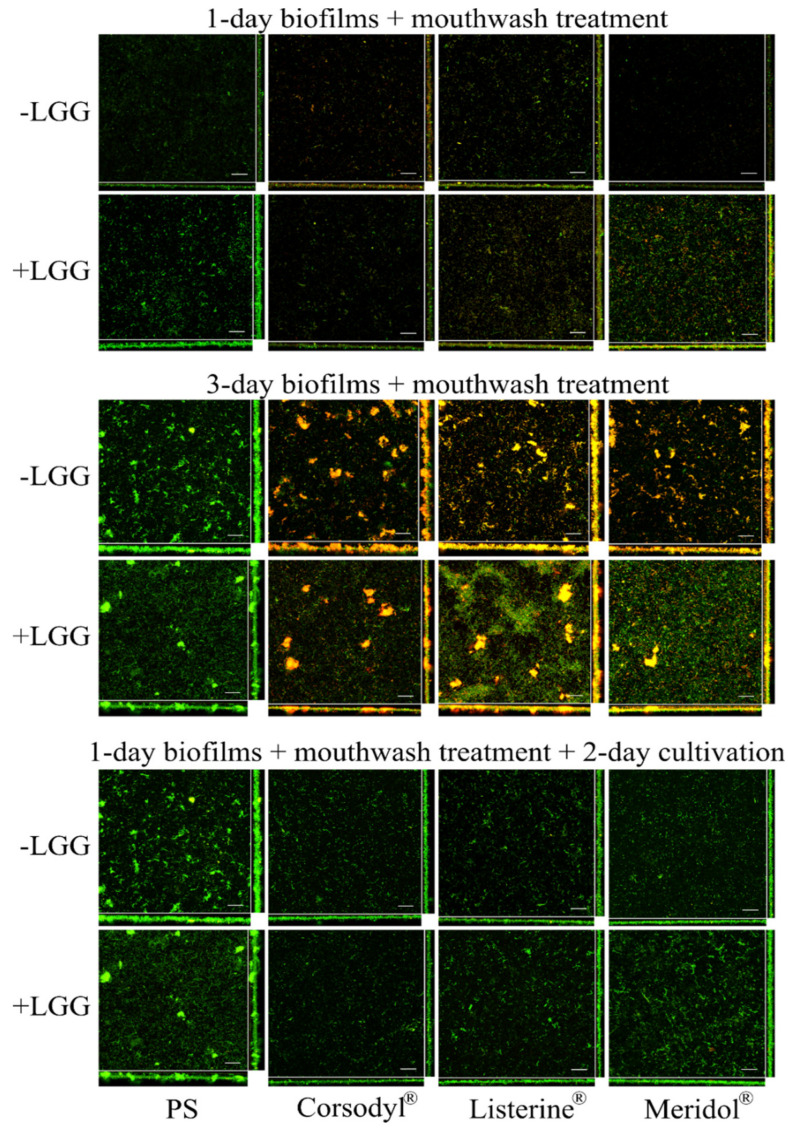
Live/dead staining of biofilms treated with the mouthwashes. LGG-free (–LGG) and LGG-integrated (+LGG) biofilms (compared). First column shows biofilms treated with physiological saline (PS), second column treatment with Corsodyl^®^, third column with Listerine^®^, fourth column with Meridol^®^. Green: live cells, red: dead cells, orange or yellow: overlap of live and dead cells. Each image includes the maximum intensity projections of xy- (center), yz- (right, rightmost is closer to HA discs), and xz-planes (bottom, bottom end is closer to HA discs). Scale bars, 30 µm.

Both live and dead cells were detectable on each HA disc in the CLSM images (Figure 4). Dead cells were obvious in 1-day and 3-day biofilms when treated with the mouthwashes, compared with the findings with PS treatment. In the 1-day biofilms, the cells grew randomly and scattered and formed thin layers. In 3-day biofilms, the cells grew in clots and clusters and formed thicker layers.

### 3.3. Recovery of Streptococci and Candida in LGG-Free and LGG-Integrated Biofilms after the Mouthwash Rinsing

#### 3.3.1. Recovery Rates of Streptococci and Candida in LGG-Free and LGG-Integrated Biofilms

**Figure 5:**
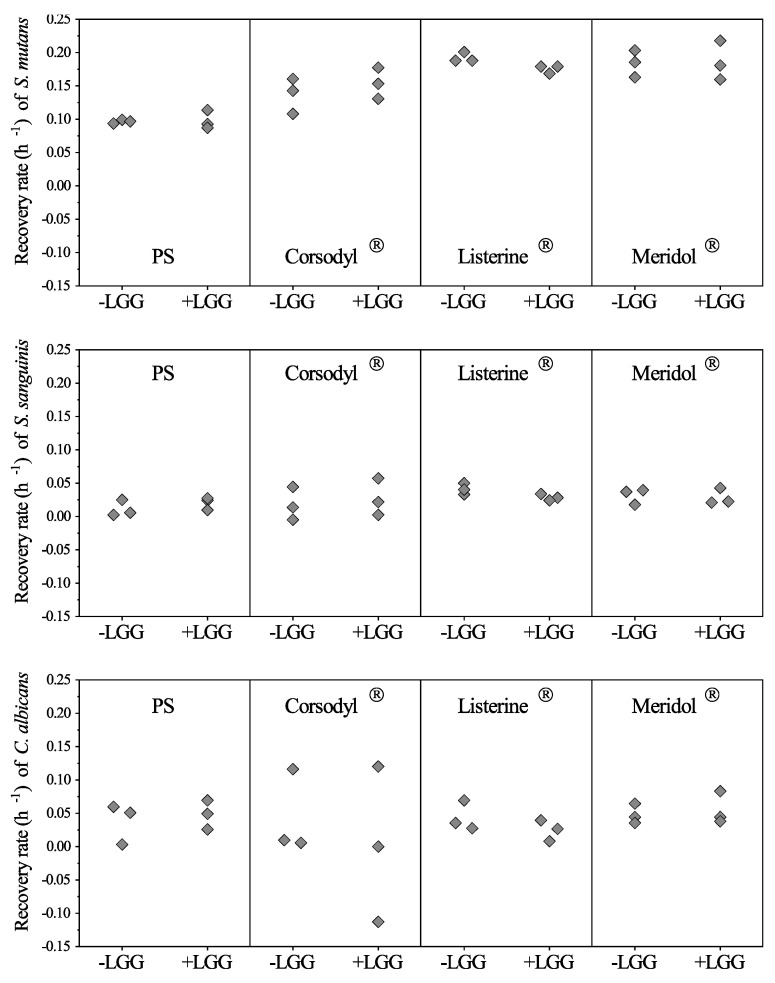
Recovery rates of the strains in LGG-free (-LGG) and LGG-integrated (+LGG) biofilms after mouthwash rinsing. Values as diamonds. The independent samples Mann–Whitney U-test revealed no significant differences (*p* > 0.05).

Between the LGG-free and LGG-integrated biofilms, no significant differences appeared in the recovery rates of each strain during the 2-day continuous cultivation after the mouthwash treatment (*p >* 0.05, Figure 5). The recovery rates of *S. mutans* were generally higher than those of *S. sanguinis* and *C. albicans*. Compared with PS, all the mouthwashes: Corsodyl^®^, Listerine^®^, and Meridol^®^ reduced the median values for the recovery rates of *C. albicans* but raised the median values for the recovery rates of *S. mutans.*

#### 3.3.2. Structural Analysis of Biofilms

Following 2-day continuous cultivation after the mouthwash treatments on 1-day biofilms, the cells grew in clots, clusters, and formed thicker layers; dead cells were rarely seen after treatment with mouthwashes (Figure 4).

## 4. Discussion

In our 1-day and 3-day multi-species test of relative survival rates of LGG and SSC in the LGG-integrated biofilms, LGG was susceptible to all the mouthwashes tested in both the 1-day and 3-day biofilms. Although able to establish itself in the biofilm structure, LGG showed no influence on streptococci and *Candida* responses to the mouthwashes and did not alter their recovery ability following the experiment.

We adhered to a biofilm model used earlier [27]. Six species were selected according to their clinical relevance: *S. mutans* and *S. sanguinis*, which are associated with dental caries; *A. actinomycetemcomitans* and *F. nucleatum*, which have been related to periodontitis; *C. albicans*, which has been associated with oral candidiasis; and LGG, one of the most studied and consumed probiotic strains. *A. actinomycetemcomitans* and *F. nucleatum* served as background strains to add to increase the complexity of the biofilm system. They both were able to integrate into the experimental biofilms, but we were unable to cultivate them from BHI agar plates [27], cultivation which was essential for the counting of *S. mutans* and *S. sanguinis* in this study.

LGG was susceptible to all of the tested mouthwashes with values insignificantly smaller than those of *S. mutans*, *S. sanguinis* and *C. albicans* in both 1-day and 3-day biofilms. The relative survival rate in this study allowed comparison of the strains’ susceptibilities to the mouthwashes. A lower relative survival rate indicates higher susceptibility to the mouthwashes. Yousefimanesh et al. evaluated the minimum inhibitory concentrations (MIC) and minimum bactericidal concentration (MBC) with the tube dilution method and found, in agreement with our own findings, that *Lactobacillus casei* was more susceptible to CHX mouthwashes than was *S. mutans* [40]. Conversely, some groups have reported that *L. acidophilus* in an agar diffusion assay [25], *L. rhamnosus* in in vitro biofilms [41,42,43], and salivary *L. rhamnosus* and lactobacilli in vivo [23,44] were less susceptible to CHX than were *S. mutans* and *S. sanguinis*. Such a difference between study results may be the result of such variables as strain type, (e.g., freshly isolated *Lactobacillus* strains from lesions of nursing caries are generally more tolerant than the type collection strains) [45,46]; as differences in species [46]; as differing concentrations of active components [46]; and as differences in component combinations [47]. Hence more studies with standardized methods may enable further conclusions in this area.

In our study, the presence of LGG in the biofilms neither raised nor reduced the efficacy of mouthwashes on the tested streptococci and *Candida*. This result may imply that the colonization of probiotic LGG in the current biofilm model did not release any metabolites that could weaken the effects of the mouthwashes on the streptococci and *Candida*.

Each of our streptococci and *Candida* were more susceptible to the mouthwashes in our 1-day biofilms than in our 3-day biofilms. This result is in agreement with findings that biofilm age affects mouthwash efficacy. For example, CHX has shown only a minor effect on the viability of established/matured biofilms in vivo and in vitro [37,48,49]. Dalleau et al. have reported that the effect of 0.06% thymol on *C. albicans* ATCC 66396 was age-related when the biofilm age was no more than 3 days [50]. Exterkate et al. have shown that saliva-derived biofilms became more resistant to amine fluoride (AmF) with age [51]. In order to interpret the experimental findings for multispecies oral biofilms, Shen et al. [52] developed a mathematical model and reported that the proportion of killed bacteria volume was a decay function of biofilm age. However, data from the study of Dalleau et al. also showed that 4- and 5-day biofilms of *C. albicans* were less resistant to 0.06% thymol than were their 3-day biofilms [50]. A 7-day *Salmonella* biofilm did not display more resistance to CHX gluconate (1 mg/mL) than did 3- or 5-day biofilms in one study, in which those conflicting results probably reflect the diverse conditions of growth, differences between the devices used in the studies, and the different surface nature of biofilm formation. Again, more studies are needed also in this area [53].

Interestingly, in the present study, some relative survival rates of the strains in 3-day biofilms after mouthwash treatment exceeded 100%. The CLSM images showed that, upon biofilm exposure, the antimicrobial effects of the mouthwashes were immediate, and the result was immediate cell death. PS treatment was regarded as causing no harm to the live cells in biofilms. Relative survival rates should, therefore, be no higher than 100%. Two possibilities may explain our result. First, the rinsing with PS in the control group may have removed the surface cells not tightly attached to the biofilms. Second, some compounds in the mouthwash, for example, CHX, may have aggregated the cells, shrunk the extracellular matrix, and made the loosely bound cells firmly attached [8,52].

During the continuous 2-day cultivation, no significant differences emerged in the recovery growth rates of these streptococci and *Candida* neither between the LGG-free nor the LGG-integrated biofilms. This result was probably related to the amount of residual LGG in the LGG-integrated biofilms after the mouthwash treatment as being far less than the required functional dose to confer any health benefit. For anyone regularly consuming probiotic LGG and gaining its benefits, it is, therefore, relevant to schedule the intake after an oral hygiene procedure and the use of a mouthwash. However, the specific retake timing needs further consideration with respect to the substantivity of mouthwashes in the oral cavity. Research has demonstrated [54,55,56] that 0.2% CHX has the highest substantivity values when compared with the values of other commercial mouthwashes. A period of 3–12 h is required for 0.2% CHX to be washed out of the mouth [9,57]. Aminabadi et al. [23] have concluded that a 24-h pretreatment with CHX could enhance LGG colonization in the mouth. The underlying reason may depend on the competitive exclusion principle. The anticipated reduction in microbial species in oral biofilms may lead to a greater increase in adhesion spots for colonization upon new intake of probiotics.

Comparing our microorganisms’ recovery rates, *S. mutans* showed far higher rates than did the rest of the strains, which is consistent with findings that the growth ratio of *S. mutans* was higher than the rest of the strains in another multi-species biofilm model [27]. The fast growth of *S. mutans* may not result from the suitable broth or other environmental factors, but from the synergistic effects in the microbial community. We noticed that after mouthwash treatment, the recovery rates of *S. mutans* increased; but median recovery rates of *C. albicans* decreased. In the current study, the prolonged effect of Corsodyl^®^ on *S. mutans* did not last longer than 2 days, however. Aminabadi et al. [23] reported their *S. mutans* count to be reduced 24 h after cessation of CHX and remaining at that level for up to 5 weeks. The difference in recovery rates between species may have been due to the other oral microbes present. In the oral cavity, in excess of hundreds of species of oral micro-organisms mutually restrict each other, thus also controlling the growth of *S. mutans*. The present study’s biofilm model involved only six strains. Thus, when the growth of the rest of the strains was restricted, *S. mutans* may have been able to use the extra nutrients in the broth, resulting in its faster recovery and strain dominance under our experimental conditions.

This in vitro study had its own limitations. The biofilm model involved a maximum of six species of reference strains. The oral cavity, however, harbors numerous bacteria and yeasts, and the phylogenetic diversity varies from person to person [58]. Moreover, an individual’s eating, drinking, and oral hygiene habits can strongly affect the activities of probiotics and of mouthwash in the mouth. These facts call for corresponding studies in clinical settings before drawing further conclusions.

## 5. Conclusions

LGG can establish itself in experimental oral biofilms. However, when challenged with commercial dentifrices, its susceptibility is similar to that of the tested oral pathogens. The presence of a probiotic in 1- and 3-day biofilms does not significantly affect mouthwash efficacy or the recovery ability of streptococci and *Candida*. Hence, our study hypothesis was confirmed, but the clinical relevance of our findings needs further assessment.

## Figures and Tables

**Table 1:** Strains and growth conditions.

Strain	Origin	Agar/Broth	Cultivation Time, Temperature, and Air Composition
*Lactobacillus rhamnosus* GG ATCC ^1^ 53103 (LGG)	Valio Ltd., Helsinki, Finland	MRS ^2^	24 h, 37 °C, 5% CO_2_
*Aggregatibacter actinomycetemcomitans* ATCC 43718	ATCC	BHI ^3^	24 h, 37 °C, 5% CO_2_
*Candida albicans* ATCC 10231	ATCC	Sabouraud	24 h, 37 °C, air
*Fusobacterium nucleatum* ATCC 25586	ATCC	Brucella	48 h, 37 °C, mixture of 0.2% O_2_, 5% CO_2_, 9.9% H_2_, 84.9% N_2_
*Streptococcus mutans* ATCC 27351	ATCC	BHI	24 h, 37 °C, 5% CO_2_
*Streptococcus sanguinis* ATCC 10556	ATCC	BHI	24 h, 37 °C, 5% CO_2_

^1^ ATCC: American Type Culture Collection. ^2^ MRS: de Man, Rogosa and Sharpe. ^3^ BHI: Brain heart infusion.

**Table 2:** Commercial mouthwashes tested in this study.

Trade Name	Main Active Component	Manufacturer
Corsodyl^®^	0.2% (or 2 mg/mL) chlorhexidine gluconate	GlaxoSmithKline, UK
Listerine^®^ Total Care	Essential oils: eucalyptol 0.092%, methyl salicylate 0.060%, thymol 0.064% and menthol 0.042%	Johnson & Johnson, UK
Meridol^®^	Amine fluoride and stannous fluoride (250 ppm F^−^)	GABA, Switzerland
